# Emergence of amoxicillin resistance and identification of novel mutations of the *pbp1A* gene in Helicobacter pylori in Vietnam

**DOI:** 10.1186/s12866-022-02463-8

**Published:** 2022-02-03

**Authors:** Trung Thien Tran, Anh Tuan Nguyen, Duc Trong Quach, Dao Thi-Hong Pham, Nga Minh Cao, Uyen Thi-Hong Nguyen, An Nguyen-Thanh Dang, Minh Anh Tran, Loc Huu Quach, Khiem Thien Tran, Nhan Quang Le, Viet Van Ung, Minh Ngoc-Quoc Vo, Danh Thanh Nguyen, Kha Dong Ngo, Trung Le Tran, Vy Thuy Nguyen

**Affiliations:** 1 Department of Surgery, University of Medicine and Pharmacy at Ho Chi Minh City, Ho Chi Minh City, Vietnam; 2 Molecular Biomedical Center, University Medical Center, Ho Chi Minh City, Vietnam; 3 Department of Internal Medicine, University of Medicine and Pharmacy at Ho Chi Minh City, Ho Chi Minh City, Vietnam; 4 Department of Genetics, University of Science, Vietnam National University Ho Chi Minh, Ho Chi Minh City, Vietnam; 5 Department of Microbiology-Parasitology, University of Medicine and Pharmacy at Ho Chi Minh City, Ho Chi Minh City, Vietnam; 6 University Medical Center – Campus 2, Ho Chi Minh City, Vietnam; 7 Department of Endoscopy, University Medical Center, Ho Chi Minh City, Vietnam; 8 Department of Oral Biology, Yonsei University College of Density, Seoul, South Korea

**Keywords:** Amoxicillin resistance, *Helicobacter pylori*, Mutation, *pbp1A* gene, Vietnam

## Abstract

**Background:**

Amoxicillin-resistant *Helicobacter pylori* (*H. pylori*) strains seem to have increased over time in Vietnam. This threatens the effectiveness of *H. pylori* eradication therapies with this antibiotic. This study aimed to investigate the prevalence of primary resistance of *H. pylori* to amoxicillin and to assess its association with *pbp1A* point mutations in Vietnamese patients.

**Materials and methods:**

Naive patients who presented with dyspepsia undergoing upper gastrointestinal endoscopy were recruited. Rapid urease tests and PCR assays were used to diagnose *H. pylori* infection. Amoxicillin susceptibility was examined by E-tests. Molecular detection of the mutant *pbp1A* gene conferring amoxicillin resistance was carried out by real-time PCR followed by direct sequencing of the PCR products. Phylogenetic analyses were performed using the Tamura-Nei genetic distance model and the neighbor-joining tree building method.

**Results:**

There were 308 patients (46.1% men and 53.9% women, *p* = 0.190) with *H. pylori* infection. The mean age of the patients was 40.5 ± 11.4 years, ranging from 18 to 74 years old. The E-test was used to determine the susceptibility to amoxicillin (minimum inhibitory concentration (MIC) ≤ 0.125 μg/ml) in 101 isolates, among which the rate of primarily resistant strains to amoxicillin was 25.7%. Then, 270 sequences of *pbp1A* gene fragments were analysed. There were 77 amino acid substitution positions investigated, spanning amino acids 310–596, with the proportion varying from 0.4 to 100%. Seven amino acid changes were significantly different between amoxicillin-sensitive (Amox^S^) and amoxicillin-resistant (Amox^R^) samples, including Phe_366_ to Leu (*p* <  0.001), Ser_414_ to Arg (*p* <  0.001), Glu/Asn_464–465_ (*p* = 0.009), Val_469_ to Met (*p* = 0.021), Phe_473_ to Val (*p* <  0.001), Asp_479_ to Glu (*p* = 0.044), and Ser/Ala/Gly_595–596_ (*p* = 0.001). Phylogenetic analyses suggested that other molecular mechanisms might contribute to amoxicillin resistance in *H. pylori* in addition to the alterations in PBP1A.

**Conclusions:**

We reported the emergence of amoxicillin-resistant *Helicobacter pylori* strains in Vietnam and new mutations statistically associated with this antimicrobial resistance. Additional studies are necessary to identify the mechanisms contributing to this resistance in Vietnam.

## Introduction

*Helicobacter pylori* (*H. pylori*) is a Gram-negative microaerophilic pathogenic bacterium that colonises the human gastric mucosa. It has been categorised as a Group I carcinogen for gastric cancer [1]. It infects approximately 50% of individuals worldwide. In Vietnam, the prevalence of *H. pylori* is very high (75%) [2]. The rates of *H. pylori* infection in Vietnamese patients with chronic gastritis, gastric ulcers, duodenal ulcers, and gastroduodenal ulcers range from 59.9–69.9, 77.8, 85–95%, and 85.3–93.6%, respectively [3]. The incidence of gastric cancer in Vietnam has been reported to be the highest compared to other southeast Asian countries [4]. Successful *H. pylori* eradication has been demonstrated to not only heal peptic ulcers but also prevent their recurrence and reduce the incidence of gastric cancer [3, 5].

Amoxicillin is one of the key antibiotics often used to eradicate *H. pylori* in standard triple therapy (amoxicillin, clarithromycin, and proton pump inhibitor) as the first-line treatment [6]. However, the spread of amoxicillin- and/or clarithromycin-resistant *H. pylori* has become an important cause of eradication failure. A high rate of clarithromycin resistance of *H. pylori* (72.6%) has been well documented in Vietnam [7]. Additionally, recent data in Vietnam have shown that the amoxicillin resistance rate of *H. pylori* differs across regions and it seems to have increased over time, from 1.1% in 2015 to 10.4% in 2016 and 15% in 2018 [3, 8, 9]. This threatens the effectiveness of *H. pylori* eradication therapies with these antibiotics.

Point mutations in the genes coding for penicillin-binding proteins (PBPs) lead to a decreased affinity for the drug and result in amoxicillin resistance [10, 11]. Among these genes, the *pbp1A* gene seems to be one of the keys [12–15]. There have been several studies on the molecular antibiotic resistance of *H. pylori* in Vietnam, but most of them focused on clarithromycin and levofloxacin resistance [7, 9, 16]. This is the first study conducted to investigate the molecular mechanism of amoxicillin resistance in *H. pylori* in Vietnam.

The present study aimed to evaluate the prevalence of amoxicillin-resistant *H. pylori*-positive gastric biopsy specimens and to investigate point mutations in the *pbp1A* gene in *H. pylori*-sensitive and primary-resistant samples. This study contributes to uncovering novel point mutations in the *pbp1A* gene underlying this resistance effect.

## Materials and methods

### Patient population and clinical specimens

This study was conducted between January 2019 and April 2021 at the University Medical Center, Ho Chi Minh City, Vietnam. A total of 308 Vietnamese patients with positive *H. pylori* infection by rapid urease test, presenting with naive dyspepsia and aged ≥18 years were recruited. The exclusion criteria included patients with gastric cancer and/or those who took any antibiotics within the last 4 weeks.

The sample size was calculated by RaoSoft® software (http://www.raosoft.com/samplesize.html) at a 95% confidence level and alpha set at 0.05. Based on the primary amoxicillin resistance rate of 15% [3] in *H. pylori* in Vietnam, the sample size was estimated at 195 for amoxicillin resistance study. We also set up a larger sample size at the same time for studying other molecular antimicrobial resistance in *H. pylori* as well. Therefore, the sample size of 308 was finally used for the analysis of amoxicillin resistance in *H. pylori* as the result of the available data. During upper gastrointestinal endoscopy, four biopsies were obtained from each of the 308 patients recruited to the study to make sure the highest rate of *H. pylori* recovered from the sampling and to limit the influence of *H. pylori* distribution in the gastric mucosa. The first pair of specimens (one from the antrum and one from the corpus) was tested using both the rapid urease test (NK-Pylori test, Nam Khoa Biotek Co., Ltd.) and a polymerase chain reaction-based test (PCR) (*AccuPid H. pylori* Genotyping Kit, *ref. No.* Q01HPY03.1A, Khoa Thuong Biotech Co., Ltd.). The second pair was stored in transport medium [17] and kept at 2–8 °C until processed for culture within 3 h after sampling. Samples from a total of 101 patients were collected for culture. The samples can also be stored at − 80 °C in BHI (BD) supplemented with 25% glycerol until culture. The *Pbp1A* gene fragment from all samples was subjected to direct sequencing. The patient characteristics are presented in Table 1.

**Table 1:** Demographic characteristics and diagnosis of the recruited patients

Characteristics	Cases with sequenced *pbp1A* fragments	Cases with E-test and PCR
	% (*n* = 308)	% (*n* = 101)
Gender		
Male	46.1 (142)	41.6 (42)
Female	53.9 (166)	58.4 (59)
Mean age ± SD (range) (year)	40.5 ± 11.4 (18–74)	40.5 ± 11.1 (18–67)
Residency		
Ho Chi Minh	31.5 (97)	34.7 (35)
Southeast	19.5 (60)	19.8 (20)
Mekong River Delta	22.1 (68)	16.8 (17)
Central	25.3 (78)	24.8 (24)
Red River Delta	1.6 (5)	4.0 (4)
Diagnosis		
Gastritis	93.5 (288)	97.0 (98)
Gastric ulcer	6.5 (20)	3.0 (3)
E-test for amoxicillin		
Sensitive	74.3 (75)	74.3 (75)
Resistant	25.7 (26)	25.7 (26)

### Culture conditions and bacteria identification

Gastric biopsy specimens with a positive rapid urease test were collected and maintained for a maximum of 3 h in transport medium [17] until further processing. The samples were then ground in 100 μL brain heart infusion (BHI, BD) broth supplemented with 10% fetal bovine serum (FBS, Sigma–Aldrich) and cultured on Columbia agar (BD) plates supplemented with 10% lysed sheep blood (Nam Khoa Biotek Co., Ltd.), 1% isoVitaleX (BD), a skin antibiotic mixture (Sigma–Aldrich), and 2.5 μg/mL amphotericin B (Sigma–Aldrich). Plates were incubated for 4–5 days at 37 °C in a microaerobic atmosphere. A single colony of a 4–5-day-old culture was identified through morphological observation, urease tests and PCR specific to the *cagA* and *vacA* genes. A colony with specific morphology, a positive urease test and identified *cagA* and *vacA* was subcultured on Columbia agar plates as described above for 4 days. Amplification and detection of the *cagA* and *vacA* genes was performed based on a previously published protocol [18].

### Determination of the minimal inhibitory concentration

The minimal inhibitory concentration (MIC) of amoxicillin was determined by the E-test method (BioMerieux, ref.# 412243). *H. pylori* strains stored at − 80 °C were thawed on Columbia agar plates supplemented with 10% lysed sheep blood and 1% isoVitaleX and incubated at 37 °C for 3–4 days in a microaerobic atmosphere. Colonies were subcultured on another plate for 3 more days. Each plate was swabbed with a sterile cotton-tipped applicator, and the cells were suspended in BHI to obtain a turbidity equivalent to a 3.0 McFarland standard. A 100 μl bacterial suspension was inoculated on a Columbia plate containing 10% lysed sheep blood and 1% isoVitaleX, and another sterile cotton-tipped swab was used to streak in three directions across the plate. The plates were left to dry for 2–3 min, and then the E-test strips containing a continuous exponential gradient of amoxicillin were placed on the agar surface. The plates were incubated at 37 °C for 3–4 days in a microaerobic atmosphere. At the end of the incubation period, the MICs were determined by the intercept of the zones of growth inhibition with the graded E-test strip. The reference *H. pylori* strain ATCC® 43504™ susceptible to amoxicillin (Amx^S^) was always used as a quality control strain, and MIC determination was accepted only if the MIC of amoxicillin for this strain was less than or equal to 0.125 μg/ml. The susceptibility of the *H. pylori* isolates to amoxicillin was classified as resistant if the MIC was greater than 0.125 μg/ml, according to the European Committee on Antimicrobial Susceptibility Testing (EUCAST) breakpoints (https://eucast.org/clinical_breakpoints/).

### Direct sequencing of the pbp1A gene fragment

Total genomic DNA was extracted from the gastric biopsy specimens using a Qiacube automated purification system and kit (QIAGEN) according to the manufacturer’s standard instructions. Optimisation of the real-time PCR components and conditions was performed to determine the optimal conditions for the amplification of a specific fragment of the *pbp1A* gene of the bacteria in an automated thermal cycler (Roto-Gene Q, Qiagen). Sequences of the *pbp1A* primers were designed in this study as follows: pbp1A-F: 5′-CGATAGATTTGGATTACCAACGC-3′; pbp1A-R: 5′-ACGATTTCTTTACGCAAGCC-3′. The size of the expected amplicon was 1035 bp. The pbp1A-F/R primers (300 nM), MgCl_2_ (1 mM), h-Taq DNA polymerase (2 units), dNTP mix (0.2 mM), 1X PCR buffer, 1X EvaGreen and 5 μl of the extracted DNA were used in a total reaction volume of 25 μl. The optimal amplification of the target DNA was set at 95 °C for 15 min, followed by 40 cycles of denaturation at 95 °C for 30 s, annealing at 60 °C for 30 s and extension at 72 °C for 1 min. After amplification, the samples were denatured at 95 °C for 30 s and cooled to 65 °C, where they were held at that temperature for 30 s. Then, the samples were slowly heated to 95 °C at a ramping rate of 0.5 °C/s with continuous acquisition of the decline in fluorescent value. Melting curves were plotted automatically and analysed with Roto-Gene Q software. The specific melting temperature (Tm) of the *pbp1A* PCR products was 87.2 ± 0.2 °C.

The PCR products were purified with ethanol. The purified products were sequenced using a BigDye™ Terminator v3.1 Cycle Sequencing kit (Applied Biosystems, Foster City, CA). The sequencing PCR products were purified with the BigDye XTerminator™ and read in an ABI 3130 Genetic Analyser. Nucleotide sequences of both chains obtained were aligned and transformed into amino acid sequences using Geneious Prime software version 2021.1.1 (Auckland, New Zealand) and compared to the sequences of strains 26,695 (AE000511) and Hargenberg (AF479617) deposited in GenBank (http://www.ncbi.nlm.nih.gov/Genbank/) for detection of mutations in the *pbp1A* gene.

### Statistical analysis

Statistical analysis was performed using the Statistical Package for Social Science (SPSS) version 20.0. Descriptive statistical analysis was used to describe the characteristics of the patients’ gender, age, residency, gastric disease status and susceptibility to amoxicillin of the strains isolated from the clinical samples. A one-sample binomial test was used to determine whether the proportion of cases (sex, amoxicillin resistance, coinfection) was equal to the previously documented corresponding proportion. The chi-square test was used to correlate the presence of mutations in the *pbp1A* gene and the susceptibility to amoxicillin. Fisher’s exact test was used alternatively when more than 20% of the expected counts were less than 5. Monte Carlo estimates of the exact significance were used when the data did not meet the assumption of the asymptotic method. A *p*-value less than 0.05 was considered significant.

## Results

### Demographic characteristics

There were 308 qualified patients recruited in this study. Details on the demographic characteristics of these patients are presented in Table 1.

There were 46.1% (142/308; 95% CI: 40.3–51.6) men and 53.9% (166/308; 95% CI: 48.4–59.7%) women. The gender ratio occurred with probabilities of 0.5 and 0.5 (*p* = 0.190; binomial test). The mean age of the patients was 40.5 ± 11.4 (95% CI: 39.2–41.8) years, ranging from 18 to 74. Age followed a normal distribution (*p* = 0.247; skewness test). The highest percentage of patients was from Ho Chi Minh City (31.5%). The proportions of patients from nearby regions, including the southeastern area, Mekong River Delta, and central region, were similar, with proportions of 19.5, 22.1 and 25.3%, respectively; a very small percentage of patients (1.6%) were from the Red River Delta. Most patients were diagnosed with gastritis (93.5%; 288/308), and only a small percentage of them were diagnosed with gastric ulcers (6.5%; 20/308).

**Fig. 1:**
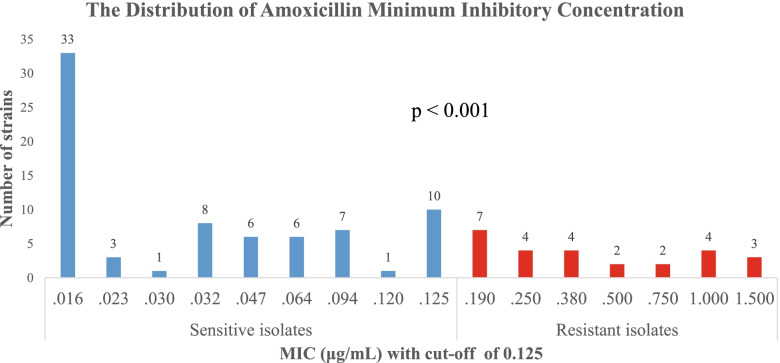
MIC range of isolates sensitive (blue) and resistant (red) to amoxicillin

Amoxicillin MIC was determined randomly in 101 samples (32.8% of the entire study group). Among these samples, 74.3% (75/101) of strains were sensitive, and 25.7% (26/101) of strains were primarily resistant to amoxicillin. The isolates with phenotypic resistance to amoxicillin exhibited a MIC range of 0.190–1.5 mg/l (Fig. 1).

### Virulence factors of *H. pylori*

The presence of the *cagA* or *vacA* gene was investigated in all 308 clinical specimens positive for the rapid urease test. Based on the *vacA* genotypes, coinfection was discovered: 38 (12.3%) specimens were colonised by at least two *H. pylori* strains (*vacA* m1 and *vacA* m2). All of the coinfection specimens were excluded from the analysis to assess the association between bacterial genotypes and clinical outcome as well as antibiotic resistance.

The *cagA* gene was present in 225 (83.3%) of the 270 *H. pylori* specimens. There was no association between gastritis and peptic ulcers with the presence or absence of the *cagA* gene (*p* = 1.000).

Regarding the *vacA* genotypes, most of the specimens had the *vacA* s1 allele (98.4%). No association was detected between the *vacA* signal sequences and the clinical outcome (*p* = 0.265). However, there was a relationship between the *vacA* middle sequences and the clinical outcome (*p* = 0.021). The *vacA* m1 allele was found to be significantly higher in 12 (75.0%) specimens from gastric ulcer patients, and the *vacA* m2 allele was significantly higher in 141 (55.5%) of the specimens from gastritis patients. The combination of the *vacA* signal and middle sequences with the clinical outcome also presented a similar significant relationship (*p* = 0.008). *VacA* s1m1 was higher in the ulcer group (75.0%), while *vacA* s1m2 was higher in the gastritis group (53.9%). The combination of *cagA* status and *vacA* genotypes was also investigated. There was an association between the combined genotypes and the clinical outcome (*p* = 0.032). In particular, no association was observed between the genotypes and the resistance to amoxicillin (*p* > 0.05) (Table 2).

**Table 2:** The genotypes of the *H. pylori* strains and the clinical outcome

Genotype	Clinical outcome			***Amoxicillin susceptibility***		
	Gastritis % (***n*** = 254)	Gastric ulcer % (***n*** = 16)	***p*** value	Resistant % (***n*** = 19)	Sensitive % (***n*** = 66)	***p*** value
*cagA*						
*cagA*^+^	83.1 (211)	87.5 (14)	1.000	78.9 (15)	80.3 (53)	1.000
*cagA*^−^	16.9 (43)	12.5 (2)		21.1 (4)	19.7 (13)	
*vacA*						
s1	98.4 (250)	93.8 (15)	0.265	94.7 (18)	97.0 (64)	0.537
s2	1.6 (4)	6.2 (1)		5.3 (1)	3.0 (2)	
m1	44.5 (113)	75.0 (12)	0.021	68.4 (13)	43.9 (29)	0.060
m2	55.5 (141)	25.0 (4)		31.6 (6)	56.1 (37)	
s1m1	44.5 (113)	75.0 (12)	0.008	68.4 (13)	43.9 (29)	0.086
s1m2	53.9 (137)	18.8 (3)		26.3 (5)	53.0 (35)	
s2m2	1.6 (4)	6.2 (1)		5.3 (1)	3.0 (2)	
*cagA* and *vacA*						
*cagA*^+^ *vacA* s1m1	42.1 (107)	68.8 (11)	0.032	63.2 (12)	40.9 (27)	0.235
*cagA*^+^ *vacA* s1m2	40.2 (102)	18.8 (3)		15.8 (3)	37.9 (25)	
*cagA*^+^ *vacA* s2m2	0.8 (2)	0.0 (0)		0.0 (0)	1.5 (1)	
*cagA*^−^ *vacA* s1m1	2.4 (6)	6.2 (1)		5.3 (1)	3.0 (2)	
*cagA*^−^ *vacA* s1m2	13.8 (35)	0.0 (0)		10.5 (2)	15.2 (10)	
*cagA*^−^ *vacA* s2m2	0.8 (2)	6.2 (1)		5.3 (1)	1.5 (1)	

### Mutational changes in or adjacent to the acyl transpeptidase conserved sequence of the pbp1A gene

**Fig. 2:**
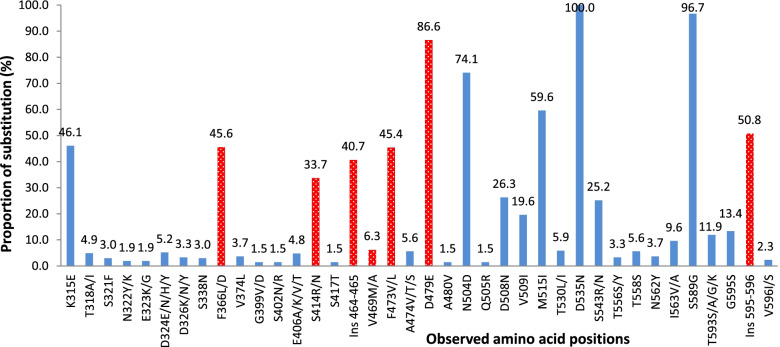
Amino acid substitution proportion of deduced PBP-1A discovered in the population compared to the Amx^S^ *H. pylori* strain 26,695. Red represents amino acid substitution differences with statistical significance from Amx^S^ and Amx^R^ samples by the E-test. A proportion lower than 1% was not included in the picture

Amoxicillin resistance in Amx^R^ *H. pylori* isolates is mediated by mutations in the *pbp1A* gene [13]. To investigate the genetic diversity of *pbp1A* genes in this study by gene sequencing, the *pbp1A* gene sequences of the *H. pylori* reference strain 26,695 (O25319, Amx^S^) and Hargenberg (AF479617, Amx^R^) were used as controls to compare and number the isolates according to the corresponding deduced amino acid sequences. A total of 270 sequences of *pbp1A* gene fragments spanning amino acids 310–596 were analysed. The results showed that the proportion of amino acid substitutions varied from 0.4 to 100% depending on the amino acid position (Fig. 2). Several amino acid positions in the acyl transpeptidase domain of PBP1A had a high proportion of substitutions, such as Asp_535_ to Asn (100%), Ser_589_ to Gly (96.7%), Asp_479_ to Glu (86.6%), and Asn_504_ to Asp (74.1%). Amino acid positions with substitution proportions equal to or lower than 1% are not shown on the graph (Fig. 2).

### Association between amino acid substitution and amoxicillin susceptibility

To investigate which *pbp1A* gene mutations were involved in amoxicillin resistance in the Amox^R^ isolates in this study, we evaluated all 77 amino acid substitutions in the acyl transpeptidase domain of PBP-1A, from amino acids 310 to 596. Seven amino acid changes were found to be significantly different between the Amox^S^ and Amox^R^ samples (Table 3), including Phe_366_ to Leu (*x*^2^ = 19.055; *p* <  0.001), Ser_414_ to Arg (*x*^2^ = 31.056; *p* <  0.001), Glu/Asn_464–465_ (*x*^2^ = 6.898; *p* = 0.009), Val_469_ to Met (*x*^2^ = 7.304; *p* = 0.021), Phe_473_ to Val (*x*^2^ = 21.223; *p* <  0.001), Asp_479_ to Glu (*x*^2^ = 4.060; *p* = 0.044), and Ser/Ala/Gly_595–596_ (*x*^2^ = 10.356; *p* = 0.001). In particular, two novel insertion mutations, Glu/Asn_464–465_ and Ser/Ala/Gly_595–596_, were discovered for the first time in this study, together with changes in Val_469_ to Met and Asp_479_ to Glu. These four novel, never-reported mutations were in or adjacent to the second (SKN_402–404_) and third (KTG_555–557_) conserved PBP motifs. The other three mutations, including Phe_366_ to Leu, Ser_414_ to Arg, and Phe_473_ to Val, have been documented [11, 13, 15].

**Table 3:** Relationships between amino acid substitution and amoxicillin susceptibility

Position	Amino acid	Genotype	Resistant % (n)	Sensitive % (n)	χ^**2**^	***p*** value
315	Glu	mt	52.9 (9)	32.8 (20)	2.312	0.128
	Lys	wt	47.1 (8)	67.2 (41)		
366	Leu	mt	78.9 (15)	24.2 (16)	19.055	< 0.001
	Phe	wt	21.1 (4)	75.8 (50)		
406	Ala	mt	5.3 (1)	4.5 (3)	0.017	1.000^*^
	Glu	wt	94.7 (18)	95.5 (63)		
414	Arg	mt	78.9 (15)	13.6 (9)	31.056	< 0.001^*^
	Ser	wt	21.1 (4)	86.4 (57)		
417	Thr	mt	0.0 (0)	1.5 (1)	0.291	1.000^*^
	Ser	wt	100 (19)	98.5 (65)		
Ins 464–465	Glu/Asn	mt	57.9 (11)	25.8 (17)	6.898	0.009
	–	wt	42.1 (8)	74.2 (49)		
469	Met	mt	21.1 (4)	3.0 (2)	7.304	0.021^*^
	Val	wt	78.9 (15)	97.0 (64)		
473	Val	mt	84.2 (16)	25.8 (17)	21.223	< 0.001^*^
	Phe	wt	15.8 (3)	74.2 (49)		
474	Val	mt	0.0 (0)	7.6 (5)	1.529	0.583^*^
	Ala	wt	100 (19)	92.4 (61)		
479	Glu	mt	68.4 (13)	87.9 (58)	4.060	0.044
	Asp	wt	31.6 (6)	12.1 (8)		
504	Asn	mt	73.7 (14)	80.3 (53)	0.387	0.534
	Asp	wt	26.3 (5)	19.7 (13)		
508	Asn	mt	31.6 (6)	24.2 (16)	0.414	0.520
	Asp	wt	68.4 (13)	75.8 (50)		
509	Ile	mt	31.6 (6)	18.2 (12)	1.586	0.208
	Val	wt	68.4 (13)	81.8 (54)		
515	Ile	mt	52.6 (10)	62.1 (41)	0.554	0.457
	Met	wt	47.4 (9)	37.9 (25)		
543	Arg	mt	26.3 (5)	25.8 (17)	0.002	0.961
	Ser	wt	73.7 (14)	74.2 (49)		
556	Ser	mt	0.0 (0)	4.5 (3)	0.895	1.000^*^
	Thr	wt	100 (19)	95.5 (63)		
562	Tyr	mt	15.8 (3)	3.0 (2)	4.338	0.072^*^
	Asn	wt	84.2 (16)	97.0 (64)		
589	Gly	mt	100 (19)	93.9 (62)	1.208	0.571^*^
	Ser	wt	0.0 (0)	6.1 (4)		
593	Ala	mt	21.1 (4)	18.2 (12)	0.080	0.784^*^
	Thr	wt	78.9 (15)	81.8 (54)		
595	Ser	mt	15.8 (3)	18.2 (12)	0.058	1.000^*^
	Gly	wt	84.2 (16)	81.8 (54)		
Ins 595–596	Gly/Ser/Ala	mt	73.7 (14)	32.3 (21)	10.356	0.001
	–	wt	26.3 (5)	67.7 (44)		
596	Ile/Ala	mt	5.3 (1)	1.5 (1)	0.878	0.403^*^
	Val	wt	94.7 (18)	98.5 (64)		

Coinfection samples were excluded from the analysis; (^*^) Fisher’s exact test was applied; (wt) wild-type; (mt) mutant (amino acid is different from strain 26,695)

We noticed that the previously described amino acid changes, such as Glu_406_ to Ala (*x*^2^ = 0.017; *p* = 1.000), Ser_417_ to Thr (*x*^2^ = 0.291; *p* = 1.000), Thr_556_ to Ser (*x*^2^ = 0.895; *p* = 1.000), and Asn_562_ to Tyr (*x*^2^ = 4.338; *p* = 0.072), associated with a high level of beta-lactam resistance in acquired multidrug-resisting *H. pylori* [19] were insignificant in this population (Table 3). Additionally, other novel amino acid substitutions mentioned previously [13] in selected Amx^R^ transformants comprising Thr_540_ to Ile, Ser_542_ to Arg, Thr_555_ to Ser, and Asn_561_ to Tyr were unchanged in this study. The results showed that several mutated positions had statistical significance between amoxicillin-sensitive and amoxicillin-resistant samples and were completely different from amino acid positions reported to be related to amoxicillin resistance published previously [15]. These mutations are adjacent to the second and third PBP motifs, which were observed only in Amx^R^ *H. pylori* colonies after transformation [13].

### Phylogenetic analyses of the pbp1A sequences

**Fig. 3:**
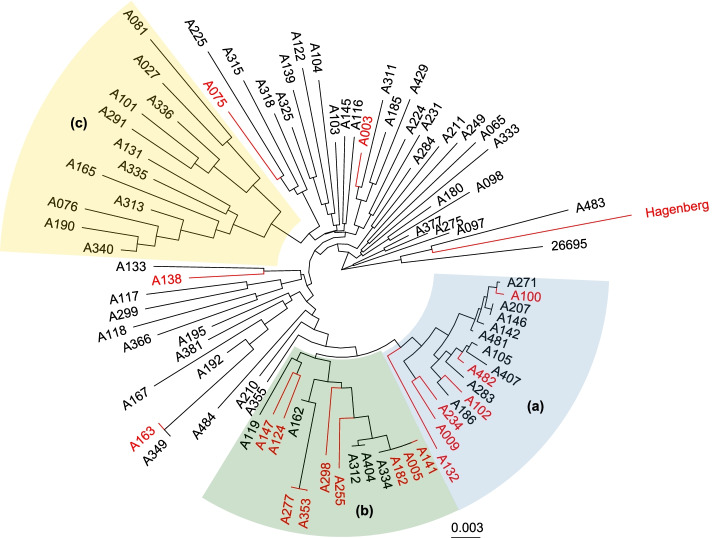
Phylogenetic tree of 85 sequences of the *pbp1A* gene obtained in this study. Scale bars indicate the numbers of nucleotide substitutions per site. Red nodes/tips represent *pbp1A* nucleotide sequences from the clinical specimens in which *H. pylori* isolates showed resistance to amoxicillin

Phylogenetic analyses were performed on all *pbp1A* gene fragment sequences to determine whether the sequences with identified mutations were in the same group of sequences related to amoxicillin resistance. Trees were constructed from 85 sequences based on the *pbp1A* amplification fragments trimmed to 879 bp by the Tamura-Nei genetic distance model and the neighbour-joining tree building method (Geneious Prime® 2021.1.1). This primary phylogenetic analysis suggested that the *pbp1A* sequences obtained in the present study might belong to different sequence groups. The sequences from which *H. pylori* isolates showed resistance to amoxicillin could be combined into two groups with close relatedness to those from which *H. pylori* isolates were sensitive to amoxicillin, except for samples A003, A075, A138 and A163. These two groups (a, b) also shared the most recent common ancestor of the *pbp1A* gene (Fig. 3). Group (c) including sensitive strains was also created as a control group, along with 26,695 and Hagenberg strains.

Table 4 shows that groups (a) and (b) had very similar amino acid substitution and insertion profiles and were quite different from the reference sequences at the seven observed positions. There were four novel strains from our samples presenting single amino acid substitutions at Leu_366_ and Val_473_, as well as amino acid insertions at Glu/Asn_464–465_ and Ser/Ala/Gly_595–596_. The other two amino acid replacements (Met_469_ and Glu_479_) also appeared uniquely in our data when compared to the references. However, the presence of specific substitutions and insertions was present not only in the resistant samples but also in the sensitive samples. For example, Met_469_ appeared in just two sensitive strains (MIC values were equal to 0.016 and 0.023 μg/mL). Leu_366_ and Val_473_ also appeared in sensitive strains, but not the sensitive ones with MIC ≤0.016 μg/mL.

**Table 4:** Overview of amino acid differences in PBP1A proteins occurring in clinical samples with resistance and sensitivity to amoxicillin

Sequence groups	E-test for Amoxicillin^b^	Clinical samples	Amino acid position^a^						
			366	414	Ins1^c^	469	473	479	Ins2^d^
Group (a)	Sensitive (0.047–0.125)	A283, A407, A105, A186, A146, A207, A271, A142, A481	Leu	Ser Arg	Glu	Val	Val	Glu	Gly Ser
	Resistant (0.190–1.000)	A132, A009, A234, A482, A102, A100	Leu	Ser Arg	Lys Glu -	Val Met	Val	Glu Asp	Gly Ser Ala
Group (b)	Sensitive (0.032–0.125)	A119, A312, A404, A334	Leu	Ser Arg	Glu	Val	Val	Glu	Gly Ser
	Resistant (0.190–2.000)	A147, A124, A277, A353, A162, A298, A255, A005, A141, A182	Leu	Arg	Lys Glu -	Val Met	Val	Glu Asp	Gly Ser Ala -
Group (c)	Sensitive (0.016–0.125)	A076, A101, A190, A291, A313, A336, A340, A131, A165, A081, A335, A027	Phe Leu	Ser	–	Val	Phe Val	Glu	Gly -
26,695	Sensitive	–	Phe	Ser	–	Val	Phe	Asp	–
Hagenberg	Resistant	–	Phe	Arg	–	Val	Phe	Asp	–

^a^ Positions of amino acid differences are given to the relative start point of the *pbp1A* gene of *H. pylori* Hagenberg (AF479617); ^b^ mg/l; ^c^ Amino acid inserted between positions 464 and 465; ^d^ Amino acid inserted between positions 595 and 596

## Discussion

Amoxicillin resistance in *H. pylori* is one of the greatest concerns of clinicians because anti-*H. pylori* regimens often consist of amoxicillin in addition to other antibiotics and proton pump inhibitors [20, 21]. Recent studies have suggested that amoxicillin resistance in *H. pylori* results from alterations in PBP1A [12, 13, 15, 22]. One of the main purposes of this study was to investigate the molecular mechanism of amoxicillin resistance in *H. pylori* strains collected from endoscopic biopsies in Vietnam.

The amoxicillin-resistant *H. pylori* proportion discovered in this study (25.7%) was similar to the finding by Saniee et al. in Iran (27.1%; *p* = 0.423) in 2018 [23] but significantly higher than that in other studies, such as Zerbetto et al. in Argentina (7.6%, *p* < 0.001) in 2017 [15], Manal et al. in Egypt (18.3%; *p* = 0.035) in 2018 [24], Ortis et al. in Central America (10%; *p* < 0.001) in 2019 [25], Azzaya et al. in Mongolia (11.9%; *p* < 0.001), Aumpan et al. in Cambodia (0%; *p* < 0.001) in 2020 [26, 27], Calinga-Ponce et al. in Mexico (1.8%; *p* < 0.001) and Li et al. in China (0%; *p* < 0.001) in 2021 [28, 29]. Compared to previous studies in Vietnam, the amoxicillin resistance proportion has been trending up significantly, for instance, from 0% (*p* < 0.001) in 2013 [30], 1.1% (*p* < 0.001) in 2015 [8], 10.4% (*p* < 0.001) in 2016 [9], 15% (*p* = 0.002) in 2019 [3] and to 25.7% in 2020 for this study. Although the previous local studies were different from each other regarding geographical areas, sample sizes, study period and antimicrobial testing methods, an increasing proportion of amoxicillin resistance in *H. pylori* in Vietnam has been generally demonstrated, suggesting that this is a serious emerging threat to the success of amoxicillin-based regimens. The high rate of amoxicillin-resisting *H. pylori* in our study might be explained by the fact that the combination of amoxicillin and clavulanate potassium is often empirically prescribed for various infectious diseases [31]. The incorrect use of antimicrobials can accelerate the selection of drug-resistant strains [23].

Multiple strains of *H. pylori* can coinfect the same patient [32]. The coinfection detected by *vacA* genotype in our study presented a rate of 12.3%. A similar proportion in terms of coinfection determination was obtained when confirmed by random amplified polymorphic DNA (RAPD) fingerprinting (12.5%; *p* = 0.500) [32] or through *vacA* and *iceA* genotyping (11.0%; *p* = 0.255) [33]. Therefore, coinfection needs to be excluded before evaluating the relationship between *H. pylori* genotypes and other factors, such as disease status and clinical symptoms, to maintain accuracy. Moreover, coinfection could undermine the success of eradication therapy and should be considered when interpreting the results of antimicrobial susceptibility tests [32].

Based on the *H. pylori* genotype analysis, our data showed an association between the *vacA*^m1^ genotype and gastric ulcers. This result suggests that individuals colonised with *vacA*^m1^-positive *H. pylori* strains are at an increased risk of developing gastric ulcers. These results confirmed the *vacA*^*m1*^ genotype is associated with an increased risk of peptic ulcers, which has been reported by Nguyen et al. [34] and Trang et al. [35] in Vietnam. Conversely, Milad et al. revealed that the *vacA*^*m2*^ genotype was significantly higher in patients with peptic ulcer disease than in patients with gastritis in Iran [36]. On the other hand, several previous studies published by Godoy et al. in Brazil and Loivukene et al. in Estonia did not find any association between virulence factors such as *vacA*^m1^ genotype and clinical outcomes or bacterial resistance to metronidazole, although the coinfection by multiple strains has been well considered in these studies [33, 37]. The difference in *cagA* and *vacA* genotype proportions as well as the association of these genotypes with the clinical outcomes might result from the fact that various populations have been evaluated with different ethnic groups and that a high genetic variability of strains in different countries exists [38, 39]. However, there is universal agreement regarding the role of the *vacA*^*m1*^ genotype among studies carried out in Vietnam at different time points. In addition, we did not find any association between the virulence factors and resistance to amoxicillin.

Amino acid substitutions in the acyl transpeptidase domain of PBP1A are required for resistance to amoxicillin [12, 13], especially F473L alteration recently discovered to be the important genetic determinant of resistance to amoxicillin of *H. pylori* in Cambodia [40]. However, other mechanisms could be involved in increasing the MIC value and contributing to the levels of high Amx^R^ strains, such as decreasing membrane permeability due to altered porin proteins (HopC, HopH), increasing the activity of efflux pumps to eject antibiotics from the periplasm, or even reducing the binding of antibiotics to other PBPs, especially PBP2 [19, 41].

In this study, we discovered a high rate of resistance to amoxicillin. To identify the mutations of *pbp1A* responsible for amoxicillin resistance, sequences of the *pbp1A* gene were analysed. We found seven amino acid changes possibly linked to amoxicillin resistance in clinical samples. Among them, the Ser_414_ to Arg substitution has been proven to be the main factor in amoxicillin resistance of the Hardenberg strain by site-directed mutagenesis [12] and it was also common in clinical Amx^R^ strains by natural transformation [11, 13]. The Phe_366_ to Leu alteration was reported to be present in the clinical Amx^R^ strain SZ79 in combination with the Ser_414_ to Arg substitution [13]. Phe_473_ was recognised in strains sensitive to but less susceptible to amoxicillin but also in Amx^R^ transformants in the absence of Ser_414_ to Arg substitutions [15], while Val_473_ was common in our Amx^R^ samples in the context of Ser_414_ to Arg changes. Although it has become clear that amino acid variations conferring resistance vary by the geographical origins of the strains [15], these data have confirmed the combination of amino acid substitutions or mutations in multiple loci to amoxicillin resistance [13].

To investigate the relatedness, as well as the combination of different mutations of *pbp1A* sequences in the resistance to amoxicillin, we created a phylogenetic tree of 85 obtained *pbp1A* gene fragments. The grouping of the *pbp1A* gene sequences suggested that there were other mechanisms in addition to the mutations in the *pbp1A* gene contributing to amoxicillin resistance in *H. pylori* in Vietnam. Other studies have also shown that distinct mechanisms of antimicrobial resistance also play important roles in the resistance to amoxicillin in *H. pylori* [13].

Our study had several limitations. First, resistant mutations were not identified directly from isolates but only from gastric biopsy specimens. We excluded coinfection cases from the data analysis, and this approach might not be ideal for identifying the molecular mechanisms related to amoxicillin resistance. Second, the role of other genes that could have synergistic effects in amoxicillin resistance could not be excluded. We did not evaluate coinfection by multiple *H. pylori* strains by fingerprinting methods such as random amplified polymorphic DNA (RAPD) or multilocus sequence typing (MLST). Last but not least, resistance has not been confirmed to have a direct correlation with eradication efficacy in real-life practice.

However, the strength of this study is that it was conducted at one of the largest hospitals in southern Vietnam, which usually admits patients from Ho Chi Minh City and many nearby areas. This is the first study that reported mutations related to Amx^R^ *H. pylori* in Vietnamese patients and it has identified some novel mutations, especially insertion mutations Glu/Asn_464–465_ and Ser/Ala/Gly_595–596_ in the *pbp1A* gene and other nearby mutations, which are likely specific to *H. pylori* strains in Vietnamese. Further studies are required to validate the role of these novel mutations in conferring amoxicillin resistance. In addition to the emerging prevalence of amoxicillin-resistant *H. pylori* strains, direct detection of *pbp1A* gene mutations from *H. pylori*-positive biopsy specimens may lead to novel diagnostic strategies for amoxicillin resistance determination and would be useful in clinical practice. More importantly, other mechanisms, such as the acquisition or expression of β-lactamase or changes in other proteins involved in cell wall synthesis, such as PBP2, PBP3, and PBP4, should also be evaluated.

## Conclusion

Our study has identified new mutations that are statistically associated with amoxicillin resistance and demonstrated the importance of the detection of amoxicillin-resistant *Helicobacter pylori* in clinical practice because of the emergence of these strains in Vietnam. However, further studies should be carried out to identify additional mechanisms contributing to amoxicillin resistance in *H. pylori*.

## Data Availability

The detail data and materials available on request (anh.nt@umc.edu.vn).
